# Consumption patterns and attitudinal insights on artificial sweeteners in Saudi Arabia

**DOI:** 10.3389/fpubh.2025.1643309

**Published:** 2025-07-16

**Authors:** Buthaina M. Aljehany, Eman A. Abduljawad, Fadhah Alatwi, Nada Benajiba

**Affiliations:** ^1^Food and Nutrition Department, Human Sciences and Design Faculty, King Abdulaziz University, Jeddah, Saudi Arabia; ^2^Regional Designated Center of Nutrition, Rabat, Morocco

**Keywords:** artificial sweeteners, consumption patterns, attitudes, risks, benefits, Saudi adults

## Abstract

**Introduction:**

Artificial sweeteners (AS) are increasingly used as sugar substitutes in Saudi Arabia, yet no studies have examined the patterns and attitudes related to their consumption.

**Aim:**

To investigate AS consumption behaviors and attitudes among Saudi adults.

**Methods:**

A cross-sectional study was conducted among 386 Saudi adults using a validated online questionnaire, which included the Artificial Sweeteners Attitudes Scale (AASS) to assess acceptance, risk perception, perceived benefits, trust in regulators, and motivation for natural alternatives. Descriptive statistics summarized consumption patterns, and chi-square tests, regression analyses, and a hurdle model were applied to identify predictors of AS use.

**Results:**

About 42% of participants reported regular AS consumption, with 65% using AS primarily for weight management and 74.19% believing AS supports a healthy lifestyle. Education level was significantly associated with AS use (*p* = 0.001). While 61.40% expressed acceptance of AS, concerns about health (44.30%) and a preference for natural foods (66.84%) remained. Frequency of AS consumption was significantly associated with AASS subscales including acceptance, perceived benefits, health risk perception, and preference for natural alternatives. Female gender (OR 1.8, 95% CI 1.2–2.6) and higher education (OR 1.5, 95% CI 1.1–2.1) emerged as significant predictors.

**Conclusion:**

This study highlights a high prevalence of AS use among Saudi adults, largely motivated by weight management goals but tempered by health concerns and a preference for natural foods. These findings underscore the need for targeted educational and public health interventions to support informed dietary choices.

## 1 Introduction

The increased tendency among consumers to reduce sugar intake has led to a broader availability of food products containing artificial sweeteners (AS) (1). AS are substances that mimic the flavor profile of sugar and are extensively used as food additives (2). These molecules are increasingly employed in food and beverage reformulation to reduce sugar content, providing a sweet taste with minimal caloric impact (3). The Food and Drug Administration (FDA) has approved the use of six AS: Acesulfame-K, Aspartame, Advantame, Neotame, Saccharin, and Sucralose. Each sweetener has an Acceptable Daily Intake (ADI) established by the FDA, with daily maximums as follows: 15 mg/kg body weight for Acesulfame-K, 50 mg/kg for Aspartame, 32.8 mg/kg for Advantame, 0.3 mg/kg for Neotame, and 5 mg/kg for both Saccharin and Sucralose (4). However, concerns about the safety of AS persist. Hence, a systematic review has explored their relationships with cardiometabolic health issues, including weight gain, metabolic syndrome, and type 2 diabetes (5).

Regarding consumption, analysis of five National Health and Nutrition Examination Survey (NHANES) cycles between 1999 and 2008 showed that 30% of US adults used some form of AS, with 19% consuming sweetener-containing beverages, 11.4% using tabletop sweeteners, and 4.6% consuming foods containing sweeteners (6). From 2007 to 2012, another NHANES-based study revealed that 47.8% of US individuals consumed multiple AS products over 2 days (6). The 2012 International Food Information Council Foundation survey found that 51% of American adults reduced sugar intake by consuming AS, while 44% aimed to avoid high-fructose corn syrup, and 29% preferred low-calorie sweeteners (7). In the UK, research indicated divided beliefs about AS: 25% of surveyed individuals perceived them as harmful, with lower intake linked to higher perceived risks. Educational interventions to increase awareness of AS’s health benefits have been shown to reduce negative perceptions (8).

In Saudi Arabia, the Saudi Food and Drug Authority (SFDA) regulates the use of artificial sweeteners in food and beverage products, in alignment with international standards such as those set by the Codex Alimentarius. The SFDA specifies permissible levels and approved types of sweeteners used in locally marketed products, ensuring consumer safety and product compliance (9, 10). From consumer perspective, Gowdar et al. (11) reported that 60.2% of participants were familiar with sugar substitutes (11). In Jeddah, Bouges et al. (12) identified Acesulfame-K as the most prevalent sugar substitute in sugar-free products, accounting for 64% (12). Alharthi et al. (13) further explored consumption patterns in the Tabuk region, revealing significant associations between AS use and demographic factors, including age, gender, and health status. However, this study did not examine attitudes or preferences across different regions of Saudi Arabia (13).

Although these studies shed light on the availability and use of artificial sweeteners in Saudi Arabia, a critical gap persists: no national-level study has yet assessed consumption patterns and attitudes across the diverse socio-demographic and geographic profiles within the country. This study aims to bridge these gaps by examining consumption behaviors and attitudes toward AS among Saudi adults, providing evidence-based findings to guide tailored health awareness campaigns.

## 2 Methodology

### 2.1 Study design and subjects

This was a cross-sectional study conducted from November 2023 to February 2024 among Saudi adults (≥18 years old) residing in the Kingdom of Saudi Arabia. The exclusion criteria included individuals currently diagnosed with eating disorders or medical conditions, such as Phenylketonuria, that could affect the consumption of AS. The study received ethical approval from the Human Research Ethics Committee of King Abdulaziz University (Reference No. 453–23). Participants were informed about the purpose of the study and their voluntary involvement through a consent form, which also provided the option to withdraw at any time. There were no risks involved in participation, no direct benefits were offered, and participant information remained anonymous. Each participant provided consent before completing the questionnaire. To ensure data quality, the online questionnaire was configured with an option that allows only one submission per participant, thereby reducing the risk of duplicates. In addition, responses were checked for completeness before inclusion in the final dataset.

### 2.2 Study sample and sampling technique

The sample size, calculated based on GASTAT’s 2022 (14) population data for Saudi Arabia, was determined assuming a 50% prevalence rate of artificial sweetener consumption, resulting in a required sample of 386 adults. This calculation utilized a formula with an effect size of 0.5 and a significance level of 0.05, determined using an electronic calculator. Data were collected using a convenience sampling technique.

### 2.3 Data collection

The questionnaire was composed of three sections as follows:

***Section 1: Socio-demographic and health data:*** This section encompassed 13 questions focusing on age, sex, place of residence, nationality, educational level, monthly family income, marital status, and health status.

***Section 2: Attitudes of artificial sweetener scale:*** The Artificial Sweetener Attitude Scale (AASS) was utilized in the study to assess participants’ attitudes towards AS. This validated and reliable scale, recently published by (15), consists of 23 items categorized into five dimensions: acceptance, risk, benefit, trust in regulators, and motivation for natural foods. The ‘Acceptance’ dimension includes 5 items that evaluate participants’ acceptance of foods containing AS, while the ‘Risk’ dimension comprises 4 items that measure participants’ perceptions of the health hazards posed by AS. The ‘Benefit’ dimension also includes 5 items and assesses whether participants perceive AS beneficial for their health. The ‘Trust in Regulators’ dimension, which is the third category, includes 3 items: “I trust the regulators (such as the Food and Drug Authority) to make sure every necessary step is taken to protect consumers’ health,” “I think that you can trust the regulators (such as the Food and Drug Authority),” and “I trust the regulators (such as the Food and Drug Authority) in relation to the licensing and control of AS in foods. “Finally, the ‘Motivation for Natural Foods’ dimension consists of 6 items and evaluates the preference for natural foods over those containing AS. Participants responded to each item on a 6-point Likert scale, ranging from 1 (very strongly disagree) to 6 (very strongly agree). The minimum score for the scale was 23, and the maximum score was 138. The total scoring for each dimension was calculated for each participant to assess their attitudes comprehensively. The questionnaire was developed and validated in English but was translated into Arabic for this study to ensure cultural relevance and accessibility for Saudi participants. It was then back translated into English by experts fluent in both languages to confirm its accuracy.

***Section 3: Sweeteners consumption frequency:*** The third section of the questionnaire was adapted from the research by Christiansen et al. (15) and focused on assessing the frequency of artificial sweetener consumption among participants (15). This section was specifically designed to quantify the number of occasions participants consumed products with AS, such as sugar-free carbonated drinks or sweetener-added beverages like tea or coffee. To ensure cultural relevance, the questionnaire was tailored to align with Saudi food habits. The time frame for the consumption data was set to the past month, with a range of frequency categories provided for participants to choose from. These categories included options like ‘never,’ ‘less than once a month,’ ‘once a month,’ ‘2–3 times a month,’ ‘1–2 times a week,’ ‘3–4 times a week,’ ‘5–6 times a week,’ ‘once a day,’ ‘2–3 times a day,’ ‘4–5 times a day,’ and ‘more than 6 times a day.’ To quantitatively analyze the data, each frequency category was assigned a numerical score. The scoring system was linear, where ‘never’ was equivalent to 0, ‘once a month’ to 1, ‘2–3 times a month’ to 2, and so on, ascending with the frequency of consumption. Higher scores on this scale indicated greater quantities of AS consumed. Based on the scoring obtained for each food/beverage item, a total scoring was calculated for each participant. The minimum total scoring was 0, and the maximum was 180. Hence, categorization was determined as follows: 0–60 for low consumption, 61–120 for moderate consumption, and 121–180 for high consumption. The online questionnaire was created using Google Forms and distributed through various channels, including social media platforms such as WhatsApp and Twitter, as well as via KAU University email. The questionnaire was translated into Arabic and then back translated into English by experts in nutrition and public health who are fluent in both languages.

### 2.4 Reliability testing

For reliability assessment, the Alpha Cronbach’s coefficient showed a high reliability (≥80) for risk (0.88), benefits (0.81); trust in regulators (0.95), and motivation for natural foods (0.80), while acceptable reliability was obtained for acceptance (0.66). Hence, the overall Alpha Cronbach’s coefficient (*α*) for the entire questionnaire was determined to be 0.68, indicating borderline acceptable internal consistency. Therefore, the questionnaire exhibited acceptable reliability, rendering it suitable for use in the study.

### 2.5 Statistical analysis

The study’s methodology for analyzing AS consumption utilized R Software version 4.1.2 for all statistical analyses. Data were presented as means and standard deviations for continuous variables and as frequencies and percentages for categorical variables. Continuous data were evaluated using the independent t-test for two-group comparisons and ANOVA followed by Tukey’s HSD test for comparisons across multiple groups. Categorical variables were analyzed using the Chi-squared test. To examine the factors influencing AS consumption, regression and hurdle models were applied, focusing on predictors of avoidance and consumption frequency. Statistical significance was set at *p*-value < 0.05.

## 3 Results

### 3.1 Socio-demographic characteristics of the study population

Table 1 summarizes the socio-demographic characteristics of the study population. A total of 386 study participants consented to participate in this cross-sectional survey. The basic characteristics of the studied population revealed that 67.62% were female, with the majority falling within the age range of 18 to 29 years (58.03%). In terms of educational background, a significant proportion of participants (76.42%) held a bachelor’s degree or higher. Participants with an income of less than 5,000 riyals constituted the largest group, accounting for 57.25%. Geographically, the Western region had the highest representation, comprising 65.54% of the sample. Approximately two-thirds of the participants (63.21%) reported being single, while nearly half (48.45%) identified themselves as students.

**Table 1:** Socio-demographic characteristics of the studied population (*N* = 386).

Variables	*N*	%
Gender		
Male	125	32.38
Female	261	67.62
Age (years)		
18–29	224	58.03
30–39	109	28.24
40–49	43	11.14
50–65	10	2.59
Educational level		
High school or less	67	17.36
Diploma	24	6.22
Bachelor’s degree	147	38.08
Master’s or PhD	148	38.34
Monthly family income (SAR)		
<5,000	221	57.25
5,000 to <10,000	70	18.13
10,000 to <20,000	70	18.13
≥20,000	25	6.48
Region		
Central	36	9.33
Western	253	65.54
Eastern	19	4.92
Northern	53	13.73
Southern	25	6.48
Marital status		
Single	244	63.21
Married	142	36.79
Employment status		
Student	187	48.45
Public sector employee	99	25.65
Private sector employee	35	9.07
Self-Employed	10	2.59
Unemployed	55	14.25

### 3.2 Patterns of consumption of AS

**Figure 1:**
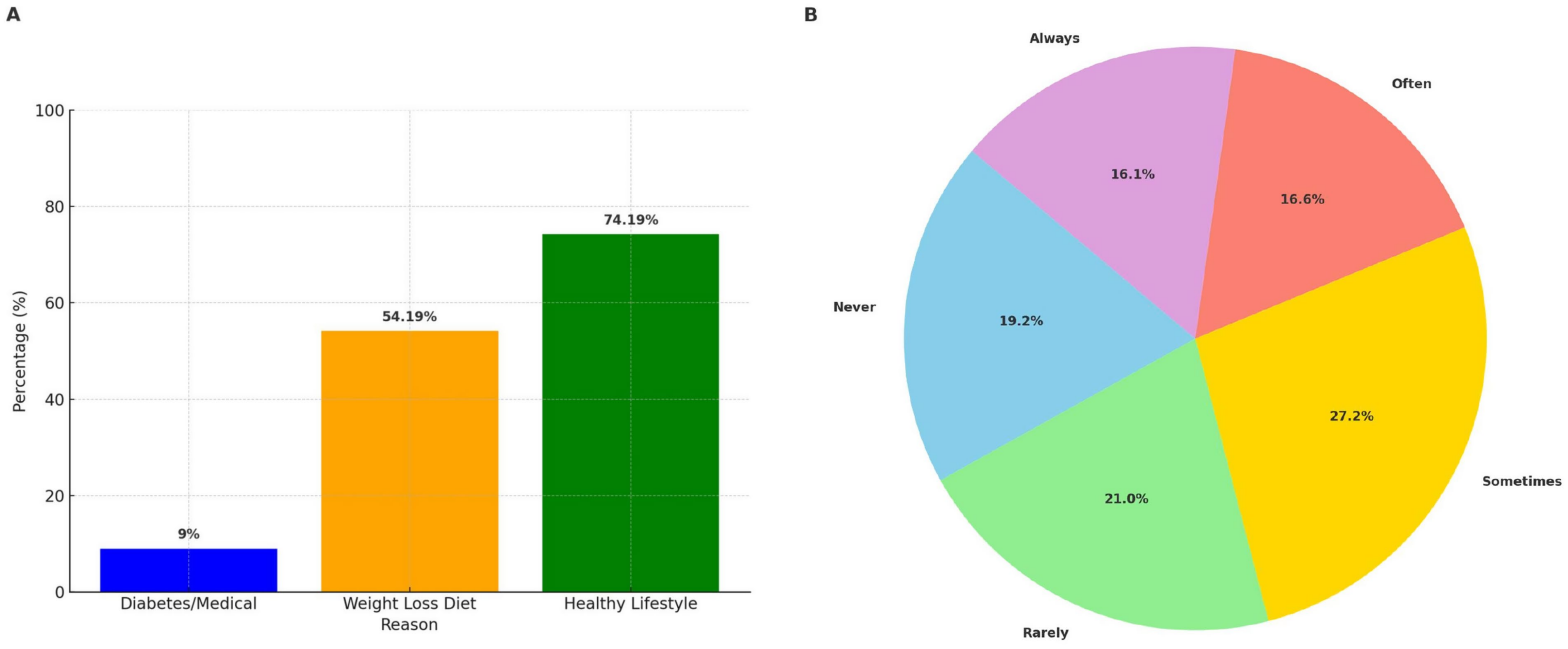
**(A)** Reasons for consuming AS (%) (*N* = 386). **(B)** Frequency of label reading for AS (%) (*N* = 386).

The patterns of AS consumption, as illustrated in Figure 1A, show that the primary reason for consuming AS is to support a healthy lifestyle, with 74.19% of participants citing this motive. This is followed by a significant proportion of participants consuming AS for weight loss purposes (54.19%). A smaller proportion of individuals (9%) reported using AS due to diabetes or other medical conditions. Regarding the frequency of reading food labels for AS content, as depicted in Figure 1B, the most common response was “Sometimes,” selected by 27.20% of participants. This was followed by “Rarely” at 21% and “Never” at 19.20%.

### 3.3 Association between frequency and levels of AS consumption and socio-demographic characteristics

The frequency of artificial sweetener (AS) consumption differed significantly across age groups, educational levels, monthly income, and regions of residence. These associations were statistically significant with *p*-values of 0.01, 0.0003, 0.014, and 0.05, respectively. Marital status also emerged as a significant factor, with unmarried individuals showing a higher likelihood of consumption compared to married individuals (*p* = 0.043). It is noteworthy that the highest consumption of food containing AS was observed in the Northern region (35.38 ± 25.81), followed by the age group of 18–29 years (31.69 ± 23.78) and unemployed individuals (31.31 ± 24.39). In contrast, the lowest consumption was recorded at 17.90 ± 21.66 among age group 50–65 years. In addition, the findings indicate that most of participants belonged to the low-level category and that there are no statistically significant differences in AS consumption across gender, age groups, income levels, marital status, and employment status (*p* > 0.05). However, an exception is observed concerning education level: a higher education level significantly correlates with increased AS consumption (*p*-value = 0.001) (Table 2).

**Table 2:** Association between the frequency and level of AS consumption and socio-demographic characteristics (*N* = 386).

Variables	Frequency of consumption (Mean ± SD)	Low (*n*, %)	Moderate (*n*, %)	High (*n*, %)	X^2^ (*p*-value)
Total		357 (92.48)	26 (6.73)	3 (0.79)	–
Gender					
Male	29.48 ± 24.32	115 (32.21)	8 (30.77)	2 (66.67)	1.626 (0.439)
Female	27.51 ± 20.33	242 (67.79)	18 (69.23)	1 (33.33)	
*p*-value	0.433				
Age (years)					
18–29	31.18 ± 23.78	201 (56.30)	20 (76.92)	3 (100.00)	7.179 (0.305)
30–39	24.30 ± 17.79	105 (29.41)	4 (15.38)	0 (0)	
40–49	24.46 ± 16.39	42 (11.76)	1 (3.85)	0 (0)	
50–65	17.90 ± 21.66	9 (2.52)	1 (3.85)	0 (0)	
*p*-value	0.01				
Educational level					
High school or less	31.69 ± 21.00	60 (16.81)	7 (26.92)	0 (0)	23.459 (0.001*)
Diploma	43.92 ± 40.16	19 (5.32)	3 (11.54)	2 (66.67)	
Bachelor’s degree	27.33 ± 19.69	33 (9.24)	2 (7.69)	0 (0)	
Master’s or PhD	24.79 ± 18.39	140 (39.22)	7 (26.92)	1 (33.33)	
*p*-value	0.0003				
Monthly family income (SAR)					
<5,000	29.87 ± 22.90	202 (56.58)	17 (65.38)	2 (66.67)	6.128 (0.409)
5,000 to <10,000	30.99 ± 21.27	63 (17.65)	7 (26.92)	0 (0)	
10,000 to <20,000	22.90 ± 19.39	67 (18.77)	2 (7.69)	1 (33.33)	
≥20,000	19.60 ± 12.26	25 (7.00)	0 (0)	0 (0)	
*p*-value	0.014				
Region					
Central	23.11 ± 15.21	36 (10.08)	0 (0)	0 (0)	
Western	28.04 ± 21.70	235 (65.83)	16 (61.54)	2 (66.67)	6.498 (0.591)
Eastern	25.47 ± 19.45	17 (4.76)	2 (7.69)	0 (0)	
Northern	35.38 ± 25.81	46 (12.89)	6 (23.08)	1 (33.33)	
Southern	23.16 ± 18.80	23 (6.44)	2 (7.69)	0 (0)	
*p*-value	0.05				
Marital status					
Single	29.75 ± 23.36	222 (62.18)	19 (73.08)	3 (100.00)	2.996 (0.224)
Married	25.39 ± 18.23	135 (37.82)	7 (26.92)	0 (0)	
*p*-value	0.043				
Employment status					
Student	30.04 ± 23.02	169 (47.34)	16 (61.54)	2 (66.67)	5.138 (0.743)
Public Sector Employee	23.64 ± 17.23	95 (26.61)	4 (15.38)	0 (0)	
Private Sector Employee	27.74 ± 21.28	33 (9.24)	2 (7.69)	0 (0)	
Self-Employed	21.40 ± 15.93	10 (2.80)	0 (0)	0 (0)	
Unemployed	31.31 ± 24.39	50 (14.01)	4 (15.38)	1 (33.33)	
*p*-value	0.096				

*p*-value calculated using *t*-test, One-way ANOVA test and, Chi^2^ test. Statistical significance set at *p*-value <0.05.

### 3.4 Attitudes towards AS in the studied population

Table 3 presents a comprehensive overview of participant attitudes towards AS, encompassing acceptability, perceived risks, benefits, trust in regulatory authorities, and preferences for natural foods. Overall, a significant majority of participants (61.40%) indicated acceptance of foods containing AS, and “*AS cannot be harmful; otherwise, they would not be contained in so many foods*” (38.08%). The highest average score (4.00 ± 1.02) was observed for the statement *“I can accept that certain foods contain AS*.” Regarding perceived risks, 44.30% of participants agreed with the statement that “*I think that certain AS are unhealthy*,” while 41.45% agreed with the statement that “*I think that certain AS are harmful to health*.” In terms of benefits, responses were more varied, with average scores generally below 4. Notably, over one-third of participants disagreed with statements asserting benefits such as “*AS allow for a reduction of unnecessary calories, bringing many benefits for consumers and allow for indulgence without regret*.” Participants demonstrated significant trust in regulatory institutions such as the Food and Drug Administration (FDA) to ensure the safety of AS, with 38.60% expressing trust. The average scores obtained for the three statements related to “trust in regulators” exceeded 4.5. Additionally, a substantial majority (66.84%) strongly agreed that natural foods are better for their health, while 62.18% strongly agreed that they feel good when they eat healthy food. Average scores for these statements were high, indicating strong beliefs (5.46 ± 0.85 and 5.37 ± 0.91, respectively) in the health benefits of natural foods.

**Table 3:** Attitudes towards AS (*N* = 386) (*n*, %).

Item	Disagree very strongly (*n*, %)	Disagree Strongly (*n*, %)	Disagree (*n*, %)	Agree (*n*, %)	Agree Strongly (*n*, %)	Agree very strongly (*n*, %)	Mean ± SD
Acceptance							
I think it is unimportant to check on the packaging whether a food contains AS.	81 (20.98)	61 (15.80)	150 (38.86)	63 (16.32)	17 (4.40)	14 (3.63)	2.87 ± 1.27
I have more important things to do than worry about AS.	44 (11.40)	47 (12.18)	107 (27.72)	134 (34.72)	28 (7.25)	26 (6.74)	3.34 ± 1.31
I can accept that certain foods contain AS.	13 (3.37)	15 (3.89)	42 (10.88)	237 (61.40)	45 (11.66)	34 (8.81)	4.00 ± 1.02
People give too much thought to AS.	26 (6.74)	48 (12.44)	161 (41.71)	112 (29.02)	29 (7.51)	10 (2.59)	3.26 ± 1.07
Artificial sweeteners cannot be harmful; otherwise, they would not be contained in so many foods.	56 (14.51)	59 (15.28)	147 (38.08)	95 (24.61)	20 (5.18)	9 (2.33)	2.98 ± 1.18
It does not bother me if my foods contain AS.	38 (9.84)	34 (8.81)	114 (29.53)	146 (37.82)	32 (8.29)	22 (5.70)	3.43 ± 1.23
Risk							
When I think of AS, I get an uneasy feeling.	36 (9.33)	33 (8.55)	119 (30.83)	139 (36.01)	33 (8.55)	26 (6.74)	3.46 ± 1.25
I am worried about what effects AS could have on my body.	20 (5.18)	24 (6.22)	84 (21.76)	140 (36.27)	53 (13.73)	65 (16.84)	3.98 ± 1.32
I think that certain AS are unhealthy.	4 (1.04)	7 (1.81)	31 (8.03)	171 (44.30)	81 (20.98)	92 (23.83)	4.54 ± 1.06
I think that certain AS are harmful to health.	6 (1.55)	6 (1.55)	49 (12.69)	160 (41.45)	66 (17.10)	99 (25.65)	2.87 ± 1.27
I think that AS are a risk to human health.	10 (2.59)	14 (3.63)	93 (24.09)	133 (34.46)	53 (13.73)	83 (21.50)	4.48 ± 1.14
Benefits							
AS allow for a reduction of unnecessary calories.	13 (3.37)	21 (5.44)	94 (24.35)	144 (37.31)	53 (13.73)	61 (15.80)	4.00 ± 1.24
AS bring about many benefits for consumers.	28 (7.25)	28 (7.25)	144 (37.31)	141 (36.53)	25 (6.48)	20 (5.18)	3.43 ± 1.13
If AS did not exist, many diet products could not be produced.	35 (9.07)	36 (9.33)	143 (37.05)	114 (29.53)	29 (7.51)	29 (7.51)	3.40 ± 1.25
The use of AS brings benefits for me personally.	55 (14.25)	28 (7.25)	144 (37.31)	122 (31.61)	18 (4.66)	19 (4.92)	3.20 ± 1.25
AS allow for indulgence without regret.	34 (8.81)	29 (7.51)	113 (29.27)	136 (35.23)	34 (8.81)	40 (10.36)	3.59 ± 1.31
Trust in regulators							
I trust the regulators (such as the Food and Drug Authority) to make sure every necessary step is taken to protect consumers’ health.	11 (2.85)	8 (2.07)	33 (8.55)	121 (31.35)	64 (16.58)	149 (38.60)	4.72 ± 1.27
I think that you can trust the regulators (such as the Food and Drug Authority).	7 (1.81)	9 (2.33)	25 (6.48)	137 (35.49)	59 (15.28)	149 (38.60)	4.76 ± 1.20
I trust the regulators (such as the Food and Drug Authority) in relation to the licensing and control of AS in foods.	7 (1.81)	6 (1.55)	37 (9.59)	140 (36.27)	62 (16.06)	134 (34.72)	4.67 ± 1.19
Motivation for natural foods							
Natural foods are better for my health.	1 (0.26)	0 (0)	7 (1.81)	61 (15.80)	59 (15.28)	258 (66.84)	5.46 ± 0.85
I feel good when I eat natural foods.	2 (0.52)	0 (0)	5 (1.30)	79 (20.47)	60 (15.54)	240 (62.18)	5.37 ± 0.91
I gladly pay a higher price for natural foods.	44 (11.40)	36 (9.33)	95 (24.61)	103 (26.68)	39 (10.10)	69 (17.88)	3.68 ± 1.54
The more natural the products are, the higher the quality of nutrients and vitamins.	5 (1.30)	2 (0.52)	8 (2.07)	74 (19.17)	60 (15.54)	237 (61.40)	5.31 ± 1.02
Natural foods taste better than other foods.	5 (1.30)	6 (1.55)	44 (11.40)	110 (28.50)	59 (15.28)	162 (41.97)	4.81 ± 1.21
I pay attention during grocery shopping to ensure that the foods are as natural as possible.	4 (1.04)	12 (3.11)	67 (17.36)	137 (35.49)	77 (19.95)	89 (23.06)	4.39 ± 1.17

### 3.5 Association between the consumption levels of AS containing foods and attitudes toward AS

Table 4 and analyze participants’ attitudes towards AS in terms of acceptability, perceived risks and benefits, trust in regulatory bodies, and preference for natural foods across different consumption levels (low, moderate, and high). The analysis shows significant variations in mean attitudes among participants based on their food containing AS consumption, as *p*-value <0.05 was obtained for all the aspects expect for trust in regulators. Acceptance of foods with AS, perceived benefits, and preference for natural foods tend to have higher mean scores among those with high AS consumption levels (26.00 ± 5.57, 23.00 ± 6.56 and 30.00 ± 6.00, respectively). Highest average of scoring as per risk perception was obtained for group of participants categorized as “low” level of consumption of foods containing AS.

**Table 4:** Attitudes according to consumption level (low, moderate, and high) of AS (*N* = 386) (Mean ± SD).

Variables	Acceptance	Risk	Benefits	Trust in Regulators	Motivation for Natural Foods
Low	19.57 ± 4.26	20.83 ± 4.93	17.42 ± 4.61	14.27 ± 3.43	29.21 ± 4.59
Moderate	22.27 ± 3.88	17.88 ± 5.23	19.65 ± 4.83	12.73 ± 4.09	26.46 ± 5.66
High	26.00 ± 5.57	20.33 ± 2.08	23.00 ± 6.56	13.00 ± 4.58	30.00 ± 6.00
P-value	<0.001*	0.014*	0.008*	0.080	0.014*

*p*-value calculated using One-Way ANOVA test. ^*^Statistical significance set at *p*-value <0.05.

### 3.6 Prediction of AS consumption using a negative binomial hurdle model

Table 5 presents the results from a negative binomial hurdle model that analyzes the predictors of AS consumption. The study considers demographic factors like gender and age, as well as attitudes such as acceptability, perceived risks and benefits, trust in regulatory bodies, and preference for natural products. The results indicate that participants with higher education levels were more likely to hold positive views toward AS and consume them more frequently, as reflected in the strong association between perceived benefits (IRR = 1.028, *p* = 0.003) and acceptance (IRR = 1.021, *p* = 0.020) with consumption frequency. AS. Moreover, individuals aged 30–39 were less likely to consume AS (IRR = 0.668, *p* = 0.021). This suggests that individuals who are more accepting of AS are more likely to consume them frequently. In addition, recognizing the benefits of AS is linked to higher consumption frequency (*p* = 0.003 and OR = 1.292). This indicates that perceived benefits play a role in influencing consumption behavior positively. While trust in regulatory authorities showed relatively high mean values, it was not a significant predictor of AS consumption.

**Table 5:** Negative binomial hurdle model predicting AS portions consumed.

Predictors		IRR	95 %CI	*p*-value
Count Model:				
Sex [F]		0.896	0.773–1.039	0.145
Age	[30–39]	0.668	0.423–1.055	0.021*
	[40–49]	0.830	0.708–0.972	0.097
	[50–65]	0.825	0.657–1.036	0.084
Acceptance		1.021	1.003–1.040	0.020*
Risk		1.005	0.988–1.022	0.592
Benefit		1.028	1.009–1.046	0.003*
Trust in regulators		0.993	0.971–1.015	0.522
Natural		0.980	0.964–0.997	0.017*
Zero Hurdle Model:		OR		
Sex [F]		1.688	0.372–7.662	0.497
Age	[30–39]	1.225	0.229–6.549	0.813
	[40–49]	1.733	0.148–20.312	0.662
	[50–65]	0.298	0.006–15.794	0.550
Acceptance		1.192	0.981–1.447	0.077
Risk		0.935	0.775–1.127	0.480
Benefit		1.292	1.030–1.622	0.027*
Trust in regulators		1.151	0.963–1.375	0.122
Natural		1.164	0.950–1.427	0.143

CI, Confidence interval; IRR, incidence risk ratio; OR, odds ratio. ^*^Statistical significance set at *p*-value <0.05.

## 4 Discussion

This aim of this present study was to explore the consumption behaviors and attitudes toward AS among Saudi adults, using a valid and reliable questionnaire. The findings provide crucial insights into the consumption of these sweeteners, emphasizing their significance in dietary practices and public health strategies. They revealed that the primary motivation for AS consumption among participants is supporting a healthy lifestyle (74.19%), followed by weight loss (54.19%). These findings align with the increasing trend of health consciousness among Saudi adults, particularly in light of Saudi Vision 2030, which promotes healthier lifestyles (16, 17). However, these results differ from prior studies (8, 18), where weight loss was the primary motivator for AS consumption. This discrepancy underscores an evolving shift in consumer behavior, emphasizing the adoption of AS part of broader health-related dietary practices.

The results also demonstrated significant variation in AS consumption across different age groups, with younger participants consuming AS more frequently (*p* = 0.01). This pattern is consistent with findings from studies in the Tabuk region and Lebanon (13, 18), where younger demographics displayed higher AS consumption. Conversely, older adults exhibited lower usage, potentially due to generational differences in dietary awareness and preferences. This contrast with findings in other regions, such as China, where children exhibited higher exposure to AS due to preferences for sweeter foods (19), suggests that cultural and regional dietary habits play a role in shaping consumption behaviors. Educational level emerged as a significant predictor of AS consumption (*p* = 0.001), indicating that individuals with higher education are more likely to consume AS. This finding aligns with previous studies (13), which suggest that education fosters awareness and acceptance of AS. However, conflicting evidence from other analyses (18, 20), highlights the need for more nuanced investigations into how educational attainment influences dietary choices. The study revealed a high acceptance of AS among participants (61.40%), with 44.30% expressing health concerns. While acceptance of AS was notable, the preference for natural foods was evident, as 66.84% of participants strongly agreed that natural foods are better for health. These findings are in line with prior research (8, 15), which highlights the duality of attitudes: while consumers recognize the benefits of AS, they remain cautious about their health implications. Notably, participants demonstrated moderate trust in regulatory bodies, with 38.60% expressing confidence in the SFDA. This level of trust underscores the importance of robust regulatory frameworks in mitigating consumer concerns and enhancing acceptance of AS. Studies suggest that trust in regulatory authorities positively impacts AS consumption (21–23), positioning these agencies as critical intermediaries in shaping public attitudes. Results of the present study also highlight that participants with a stronger preference for natural foods displayed reduced AS consumption (*p* = 0.017), highlighting the challenges faced regarding these dietary choices. Consistent with this evidence, Bearth et al. (24) found that consumers’ preferences for natural products, as well as their perceptions of risks and benefits, are crucial factors influencing the acceptance of food additives.

The application of the negative binomial hurdle model provided further depth to the analysis, revealing that acceptance and perceived benefits of AS were significant predictors of consumption (*p* = 0.02 and *p* = 0.003, respectively). This reinforces the importance of positive attitudes in driving AS usage. In fact, several studies have consistently demonstrated a robust correlation between a positive attitude and the manifestation of specific behaviors, highlighting the significant role of cognitive factors in influencing behavioral outcomes (25–27). It is worth mentioning that although participants reported a generally high level of trust in regulators, this attitude did not significantly influence actual consumption behavior in the model. This contrast may suggest that trust in regulators reflects a general belief in product safety but may not independently predict dietary behavior unless accompanied by personal relevance or health motivations.

While the study provides valuable insights, it is not without limitations. The use of convenience sampling may limit the generalizability of findings to the broader Saudi population (28). Convenience sampling can introduce selection bias, as participants who are more accessible or willing to participate may differ in important ways from those who are not, potentially affecting the representativeness of the sample (29). In fact, this type of sampling often leads to a selection bias, as participants who choose to take part may have specific characteristics or experiences that differ from those of the general population. Additionally, self-reported consumption data, which excluded precise quantities, may introduce recall bias. Future research should incorporate rigorous sampling techniques and AS intake quantification, while also considering longitudinal designs and including biochemical markers (e.g., glucose levels or BMI) to support self-reported data and build upon these findings.

## 5 Conclusion

This study highlights the multifaceted nature of AS consumption and attitudes among Saudi adults, influenced by socio-demographic factors such as education and age, with younger and more educated individuals consuming AS more frequently. Positive attitudes, particularly regarding the perceived benefits of AS, were associated with increased consumption, while a preference for natural foods showed a negative correlation. Thus, these findings underscore the need for targeted educational and public health interventions, especially for younger, more educated consumers to address misconceptions, promote balanced dietary choices, and enhance trust in regulatory bodies. They could also be utilized to guide policy development, improve AS labeling, and design targeted public health messages that support informed consumer choices. To strengthen future research, rigorous sampling techniques that ensure a more representative sample, are recommended. Additionally, quantifying AS consumption through more objective measures could improve data accuracy and reduce biases related to self-reporting. Incorporating these approaches would allow for a more comprehensive and reliable understanding of AS consumption behaviors among the Saudi population. Importantly, while this study provides valuable insights into AS consumption and attitudes, it did not explicitly apply a formal theoretical framework (such as the Theory of Planned Behavior or the Health Belief Model). Incorporating such a framework in future studies would enable a more comprehensive understanding of the behavioral and environmental factors that shape AS use within the Saudi context.

## Data Availability

The raw data supporting the conclusions of this article will be made available by the authors, without undue reservation under reasonable request.
